# Mechanisms and therapeutic potential of interactions between human amyloids and viruses

**DOI:** 10.1007/s00018-020-03711-8

**Published:** 2020-11-26

**Authors:** Emiel Michiels, Frederic Rousseau, Joost Schymkowitz

**Affiliations:** 1 VIB Center for Brain and Disease Research, Leuven, Belgium; 2 Switch Laboratory, Department of Cellular and Molecular Medicine, KU Leuven, Leuven, Belgium

**Keywords:** Protein aggregation, Antiviral, Seeding, Surface-catalyzed nucleation

## Abstract

The aggregation of specific proteins and their amyloid deposition in affected tissue in disease has been studied for decades assuming a sole pathogenic role of amyloids. It is now clear that amyloids can also encode important cellular functions, one of which involves the interaction potential of amyloids with microbial pathogens, including viruses. Human expressed amyloids have been shown to act both as innate restriction molecules against viruses as well as promoting agents for viral infectivity. The underlying molecular driving forces of such amyloid–virus interactions are not completely understood. Starting from the well-described molecular mechanisms underlying amyloid formation, we here summarize three non-mutually exclusive hypotheses that have been proposed to drive amyloid–virus interactions. Viruses can indirectly drive amyloid depositions by affecting upstream molecular pathways or induce amyloid formation by a direct interaction with the viral surface or specific viral proteins. Finally, we highlight the potential of therapeutic interventions using the sequence specificity of amyloid interactions to drive viral interference.

## Introduction

Ever since the clinical psychiatrist and neuroanatomist Alois Alzheimer reported distinctive plaques and neurofibrillary tangles in the brain of one of his patients in 1906, protein aggregates or amyloids have been widely studied in disease context [1, 2]. The main reason for this is the deposition of the amyloids in tissues that are affected by the disease. However, these amyloid depositions could still be either a cause or a consequence of the disease [2]. A century has passed and the molecular mechanisms triggering amyloid deposition and their associated toxicity are still not completely understood. However, it is now clear that amyloids per se are not necessarily toxic [3] and studies have shown the existence of functional amyloids that perform important cellular tasks, providing a structural scaffold or aiding in long-term memory consolidation [4]. Recently, it was shown that pathogenic amyloids known for their association with disease might also encode functional roles [5]. The best-studied example of this is the antimicrobial effect of disease-associated amyloids. For example, it was shown that amyloid beta, which plays a central role in Alzheimer’s Disease, binds to herpes virion particles and mediates protective antiviral effects [6]. It has even been proposed that the accumulation of amyloid in the brain of Alzheimer patients might be a direct result of this protective effect of amyloid beta against herpesvirus infections [7]. In contrast to the antimicrobial effect of amyloid beta, semen-derived amyloids promote viral infectivity, for example in case of an HIV infection [8]. In summary, many direct and indirect interactions between amyloids and viruses have been described to date. Here, we review these reported interactions between human-expressed amyloidogenic proteins and human-infecting viruses. Moreover, we provide a detailed overview of the potential mechanisms underlying these interactions that have been proposed so far. It has been well established that amyloid formation can initiate spontaneously, via surface-catalyzed nucleation or by adding preformed aggregates [9, 10]. We try to connect these three well-studied molecular processes of amyloid initiation to three potential mechanisms of amyloid–virus interactions. Finally, we discuss how amyloid–virus interactions could be exploited for the design of novel antiviral therapeutics.

### Amyloids: toxic or functional?

Protein homeostasis is a central hub in every living cell: the controlled synthesis of a specific amount of protein, the correct folding and localization, and finally the degradation of proteins are essential for cell viability [11–13]. Nevertheless, due to the complexity of protein folding, misfolding and aggregation are inevitable and are inherent to the normal functioning of a cell [14]. Protein aggregation can be the result of one of many factors, including destabilizing mutations, oxidative stress, external stress factors (e.g., heat, chemicals, etc.), or changing metabolic conditions during disease or the aging process [12, 15]. To cope with the constant pressure of protein misfolding and aggregation, cells have evolved a complex fail-safe network of pathways to ensure proper refolding or degradation of protein aggregates. This network is termed the protein quality control (PQC) system, and includes molecular chaperones, proteasome machinery, and autophagy pathways [9, 16]. Even though cells can rely on such an extensive PQC system, occasionally, protein aggregation persists and leads to harmful effects. As a result, over 35 proteins or peptides organize into protein aggregates that are associated with human diseases [9].

From the unfolded nascent polypeptide chain exiting the ribosome to the folded native state, a protein molecule can adopt different conformational states and, remarkably, even the native state is often not a highly constrained conformation, but instead resides in a precarious equilibrium [9]. This inherent instability can lead to partial protein unfolding or misfolding. Such misfolded proteins can expose sticky amino acid fragments that are usually buried inside the core of the protein [17, 18]. As a result, the misfolded proteins tend to co-assemble and form small oligomeric aggregates [19, 20]. When the aggregation reaction proceeds, oligomers can undergo substantial reorganizations to form compact β-sheet-containing structures. These preformed aggregate structures can recruit additional monomeric proteins and as such continue the aggregation reaction by self-association. The result of such aggregation reactions is the typical fibrillar architecture observed for amyloids, which are defined as protein aggregates characterized by a cross-β core [21]. The sticky amino acid fragments that compose the cross-β core of amyloids were named Aggregation-Prone Regions (APRs) and are mostly only 5–15 amino acids in length [17, 22]. It is the self-interaction of these regions that drives the amyloid reaction. Moreover, APRs strongly prefer interactions with very similar sequences, resulting in the high sequence specificity of the process of amyloid formation [23–26]. In this conformation, APRs are stapled together into β-sheets through backbone hydrogen bonds and their amino acid side chains stack laterally within the sheet and interdigitate between opposing sheets to form a tightly packed structure known as a “steric zipper” [21, 27]. This structure explains the sequence specificity of amyloid aggregation as most sequence variants would not be able to integrate with such tightly packed conformations. This unique molecular mechanism of amyloid formation results in the fact that all amyloids, formed by different aggregating proteins that bear no sequence similarity to one another, show a remarkably similar macromolecular fibril architecture.

The accumulation of over 35 proteins or peptides in well-defined amyloid states is strongly linked to human diseases [9]. Amyloid accumulation can occur in specific organs or in a systemic manner. For example, Aβ, α-synuclein, and the prion protein (PrP) form amyloid deposits in the brain, and are associated with Alzheimer’s Disease (AD), Parkinson’s Disease (PD), and Creutzfeldt–Jakob Disease (CJD), respectively, while amyloid formation of islet amyloid polypeptide (IAPP) in the pancreas is associated with type 2 diabetes (T2D) [9]. Transthyretin (TTR) and β2-microglobulin (β2-m) both lead to systemic amyloidosis by forming amyloid depositions in different organs throughout the body [9]. The origin of amyloid toxicity has been a subject of debate for many years. It appears that the soluble, oligomeric amyloid species encode the predominant toxic effect, which is most pronounced in neuropathic disorders [28–30]. However, as mature amyloid fibrils can serve as a reservoir of such oligomers and in themselves also show significant toxicity, they can hardly be labeled as inert byproducts [31]. It was shown that the exposure of hydrophobic patches in the oligomeric aggregates correlates well with toxicity. This enables the oligomers to interact with a large number of molecular species, including phospholipid bilayers, protein receptors, soluble proteins, RNAs, and small metabolites [30, 32]. Amyloid-driven interference with any of these molecular pathways can eventually lead to cell death and it seems unlikely that all amyloid-associated diseases can be explained by one unique toxic event.

In addition to this toxic gain-of-function effect of amyloids, the transformation of a native protein into an aggregated state also results in a loss-of-function effect. Most amyloidogenic proteins encode cellular functions in their native state, which are lost in their amyloid form. IAPP is a peptide hormone that plays a role in glycemic regulation [33], Aβ has a role in synaptic plasticity and memory [34] and α-synuclein is important in the regulation of neurotransmission and response to cellular stress [35]. Even more, in some cases, reducing the levels of the amyloidogenic protein in disease models is sufficient to induce disease phenotypes, even in the absence of amyloid, for example for α-synuclein [36], tau [37], and IAPP [38]. Such observations together with the fact that amyloid-like species encode cytotoxic effects complicate the untangling of the cause or consequence of protein aggregates in disease.

Due to their abundance in affected tissue of patients, for decades, human amyloids were mainly studied in disease context: amyloids were thought of as intrinsically toxic without any apparent function. In recent years, it was shown that (1) amyloids are not necessarily toxic [3] and (2) functional amyloids are present throughout all kingdoms of life [4]. In human tissue, for example, peptide hormones can organize into amyloids that act as a storage module in pituitary secretory granules [39, 40]. Even in the brain, the organ most linked to amyloid-associated diseases, an RNA-binding protein, cytoplasmic polyadenylation element-binding protein (CPEB), has been shown to organize into amyloids that are important for the consolidation of memory [41–43]. The role of the amyloid form of CPEB in long-term memory is completely independent of the RNA-binding function of monomeric CPEB. In bacteria, a protein called curli forms amyloids as an important extracellular matrix in biofilms [44]. Finally, yeast-expressed proteins such as Sup35 or Ure2p have the ability to organize into amyloid as an epigenetic non-Mendelian type of inheritance [45]. It is clear that amyloids are not just pathogenic depositions that lead to disease, and that they can actually perform important cellular functions. Moreover, for some amyloid-forming proteins that were first identified as a potential toxic agent in disease, a functional role of the amyloid form of the protein has been identified. For example, TAR DNA-binding protein 43 (TDP-43) forms amyloid aggregates in the neurons of patients with amyotrophic lateral sclerosis (ALS) [46]. However, recent evidence shows that the amyloid-like oligomeric assemblies of TDP-43 perform essential functions during regeneration of skeletal muscle in mice and humans [5]. The authors showed that these functional amyloids can also induce pathological TDP-43 amyloid fibrils leading to neuromuscular disease. Therefore, it seems that, in addition to the traditional functional amyloids that solely encode a functional role without being directly linked to disease or without any obvious pathogenic effect, disease-associated amyloids such as TDP-43 can also encode important cellular functions in their amyloid form and are not solely pathogenic byproducts.

It has been suggested that many, if not all, disease-associated amyloids may possess such functional roles in their amyloid conformation. Unraveling the precise role of amyloids might provide a new approach to combat the associated human diseases that are linked to these amyloids. The best-studied hypothesis is based on the antimicrobial properties of disease-associated amyloids [47]. This is emphasized by numerous documentations of interactions between human-expressed amyloidogenic proteins and viruses (Table 1). Here, we review direct or indirect associations between human amyloids and human-infecting viruses reported to date and discuss the potential underlying mechanisms together with the implications of these interactions. Noteworthy, amyloids have also been shown to associate with bacteria and fungi [48, 49], so, to some extent, some conclusions can be extended to the interactions between amyloids and other microbial pathogens, as well.

**Table 1:** Overview of interactions between human amyloids and human-infecting viruses

Amyloid	Virus	References
Alpha-synuclein	Influenza A	[50]
Alpha-synuclein	West Nile Virus	[51]
Amyloid beta	HSV-1	[6, 7, 52–64]
Amyloid beta	HSV-2	[65]
Amyloid beta	Varicella-zoster virus	[66, 67]
Amyloid beta	Cytomegalovirus	[68]
Amyloid beta	HHV-6A and HHV-6B	[6]
Amyloid beta precursor protein	HIV	[69]
Amyloid beta	Influenza	[70, 71]
IAPP	Varicella-zoster virus	[72]
IAPP^a^	Respiratory syncytial virus	[10]
Seminal amyloids	HIV	[8, 73, 74]
Seminal amyloids	Ebola virus	[75]
Seminal amyloids	Cytomegalovirus	[76]
Tau	HSV-1	[55, 77, 78]
Tau	HSV-2	[65]
Tau	Cytomegalovirus	[68]

^a^The interaction was shown between a short amyloidogenic fragment of IAPP and varicella-zoster virus

## Amyloid associations with viruses

Almost 3 decades ago, it was suggested that a herpes simplex virus-1 (HSV-1) infection could be an important causal agent of one of the best-studied amyloid-associated diseases: Alzheimer’s Disease [7, 79]. Since then, multiple direct and indirect interactions between human-expressed amyloids and viruses have been described. Some amyloids seem to exert a direct antiviral effect on human-infecting viruses, while others stimulate viral infection. Additionally, multiple amyloid-associated diseases have been shown to be clinically linked to viral infections without evidence of direct amyloid–virus interactions. A summary of the reported associations between human-expressed amyloids and human-infecting viruses is shown in Table 1.

### Aβ and tau interact with herpesvirus

By far the best-studied case of an interaction between amyloids and viruses is the one of HSV-1 in Alzheimer’s Disease (AD) patients [7, 52–54]. HSV-1 infects sensory neurons and via the trigeminal ganglion enters the central nervous system where it can remain in a latent state or cause acute encephalitis. It is thought that recurrent reactivation from latency over the course of years can spark the molecular mechanisms leading to AD. The first evidence linking HSV-1 to AD originated from a striking correlation between patients carrying APOE-ε4, a major genetic risk factor for AD, and the presence of herpes DNA in the brain [53]. Reversibly, APOE-ε4 was found to be a risk for cold sores, which are caused by HSV-1 [53]. A study in which 33,000 patients were monitored for 16 years showed that HSV-1 infections increase AD risk 2.5-fold [80]. Moreover, providing anti-herpetic medications reduced this risk by ~ 90%. Although the study was heavily debated [81], the results remain remarkable.

Later, evidence for direct amyloid–virus interactions accumulated. First, it was shown that HSV-1 DNA is present inside the amyloid depositions, called plaques, in the brain of deceased AD patients [55]. Moreover, Aβ, the peptide that organizes into these amyloid plaques, accumulates in HSV-1-infected cell cultures [56–58] and in the brains of HSV-1-infected mice [56]. Eimer et al. [6] showed that the presence of HSV-1 virion particles sparks amyloid formation of Aβ in 5XFAD mice and 3D human neural cell culture infection models. The authors showed that this amyloid seeding reaction was initiated by a direct interaction with the viral surface glycoproteins [6].

In addition to the direct interaction between Aβ and HSV-1, tau, the protein that forms amyloidogenic neurofibrillary tangles in AD brain, also accumulates in HSV-1-infected cell cultures [55, 77, 78]. More recently, it was shown that the herpes infections directly lead to an up-regulation of Aβ and tau and that the amyloidogenic form of Aβ actually encodes antiviral properties and directly ‘attacks’ virion particles [6, 59–62]. Interestingly, treatment with various types of antivirals such as acyclovir has been found to decrease the level of Aβ and, particularly, that of amyloidogenic phosphorylated tau [63].

In addition to HSV-1, multiple other viruses from the *Herpesviridae* family have been associated with AD. Herpes simplex virus-2 (HSV-2) infection was shown to induce AD-like neurodegeneration markers, including accumulation of hyperphosphorylated tau and Aβ [65]. Varicella-zoster virus (VZV) infection leads to an increased risk of AD by almost threefold [66, 67]. Cytomegalovirus (CMV) induces production of Aβ in cell culture and CMV serum IgG antibody levels correlate strongly with tau tangles in AD patients [68]. Human beta-herpesvirus 6A (HHV-6A) and 6B (HHV-6B) RNA levels are increased in AD brain regions, and show a correlation with amyloid plaque load and tau tangle levels. Moreover, Aβ was shown to directly bind to HHV-6A and HHV-6B surface proteins and induce amyloid formation [6].

### Aβ interacts with human immunodeficiency virus

Since the successful introduction of retroviral therapies, other conditions emerged in human immunodeficiency virus (HIV)-infected patients, mainly diseases that are associated with aging, altogether named HIV-1-associated neurocognitive disorders (HAND) [82, 83]. Amyloid plaques, reminiscent of the ones observed in AD patients, are detected in the brains of patients suffering from HAND. Moreover, it was established that HIV infection can directly lead to increased production of Aβ. It was proposed that the Aβ precursor protein (APP) may act as an innate restriction peptide that inhibits replication of HIV by sequestering the HIV Gag polyprotein in lipid rafts to block the production and spread of HIV [69]. Indeed, as most of these patients were successfully treated with retroviral therapies, limited HIV outbreaks are present. As such, the amyloid depositions found in these patients are most likely the result of direct or indirect interactions with viral proteins (e.g., Tat and Gag) instead of complete virion particles.

### Aβ interacts with influenza virus

The antiviral nature of the amyloidogenic Aβ peptide has also been shown against influenza. In vitro, it was shown that Aβ inhibits replication of seasonal and pandemic strains of H3N2 and H1N1 influenza A virus [70]. Aβ exerts its antiviral effect by inducing virion particle aggregation, thereby reducing the infection rate of this virus. The same group later showed that the C-terminal amyloidogenic fragment of Aβ is responsible for the antiviral effect against influenza A viruses [71].

### IAPP interacts with varicella-zoster virus

Amyloids formed by the short islet amyloid polypeptide (IAPP) in the pancreas is associated with type 2 diabetes (T2D). VZV-infected cells induce intracellular IAPP amyloid formation and the supernatant from VZV-infected cells induced IAPP aggregation. Interestingly, VZV glycoprotein B (gB)-derived peptides assembled into fibrils and were able to catalyze IAPP aggregation as well as Aβ-42 aggregation [72]. The latter indicates that amyloid-specific seeding events might catalyze the interaction between a viral protein and an amyloidogenic peptide.

### An IAPP peptide interacts with respiratory syncytial virus

Respiratory syncytial virus (RSV) accelerates aggregation of an IAPP-derived peptide (NNFGAIL) and this seeding effect is specific as RSV does not have an effect on the aggregation of another amyloidogenic peptide [10]. In this case, the direct interaction between an IAPP-derived peptide and RSV virion particles seems to be driven by a specific interaction instead of a specific clustering of the amyloidogenic peptide on the viral surface.

### α-Synuclein interacts with influenza

A controversial association is the one between influenza infections and α-synuclein, a protein that organizes into amyloid and deposits in dopaminergic neurons in synucleinopathies, including Parkinson’s Disease (PD). The association was first reported when postencephalitic parkinsonism appeared following the influenza pandemic of 1918–1920, also known as the Spanish flu [84, 85]. Other groups have since then also reported influenza infections as a risk factor for PD [86]. Although the possible association between influenza infections and PD is still a matter of debate, Marreiros et al. [50] have recently shown that acute H1N1 infection leads to the formation of α-synuclein aggregates in dopaminergic neurons. The authors showed that α-synuclein aggregates were induced in infected neurons connected to the olfactory bulb following intranasal infection in mice. Moreover, this α-synuclein seeding event is amyloid-specific, as no effect was observed on two additional aggregation-prone proteins: tau or TDP-43. Whether or not an influenza infection is a risk factor for PD, this study shows a direct association between infection and α-synuclein amyloid depositions, one of the major hallmarks of PD and other synucleinopathies.

### α-synuclein interacts with West Nile virus

Parkinsonism has been linked with West Nile virus (WNV) infection almost 2 decades ago [87, 88]; however, a clear association between α-synuclein and WNV is currently lacking. Interestingly, Beatman et al. [51] did show that α-synuclein inhibits WNV infection. By performing a peripheral WNV infection in α-synuclein knock-out mice, the authors observed an increased sensitivity to viral infection. Viral growth in the α-synuclein knock-out mouse brain was increased compared to wild-type mice. The authors hypothesized that α-synuclein inhibits peripheral-to-central nervous system transportation, thereby strongly reducing viral infection in the brain. They also showed that α-synuclein colocalizes with an WNV envelope protein, hinting at a direct interaction between the two.

### Semen-derived amyloids stimulate virus infection

Over a decade ago, it was demonstrated that semen harbors amyloid fibrils that drastically enhance HIV infection [8]. Currently, three different proteins are identified that can generate amyloids able to stimulate viral infection: prostatic acidic phosphatase (PAP), semenogelin-1 (SEM1), and semenogelin-2 (SEM2) [73]. In fact, it is not the full protein but rather short peptide fragments originating from these precursor proteins that organize into amyloid structures. The amyloid formed by a naturally occurring peptide comprised of residues 248–286 from PAP was found to boost the infectious titer of HIV-1 by more than five orders of magnitude, and the fibrils formed by this peptide were termed SEVI, for Semen-derived Enhancer of Viral Infection [8]. It is important to mention that the seminal amyloids are detected in HIV-1-infected as well as healthy individuals [74]. Importantly, the promoting effect observed for SEVI for enhanced HIV infection is amyloid-specific, as other amyloids, for examples those formed by the bacterial curli proteins (Csg A and CgsB) do not increase HIV infectivity [89].

In addition to HIV, other viruses can also be affected by seminal amyloids. For example, Ebola virus infection is greatly enhanced by seminal amyloids by physical interaction between the two [75]. The amyloids were able to increase viral infectivity and enhance viral stability after extended incubation at increased temperature.

Finally, in vitro experiments showed that both human and murine CMV infection was strongly enhanced by seminal amyloid in cell culture [76]. Seminal amyloids increased infection rates by more than tenfold, while replication was increased up to 100-fold. The authors showed that a physical interaction between the amyloids and the glycoproteins on the surface CMV virion particles causes this effect [76].

### Clinical associations between amyloid-associated diseases and viral infections might point to additional amyloid–virus interactions

An association between type 2 diabetes (T2D) and a hepatitis C virus infection was first described in 1994 [90]. The study showed that in a population of cirrhotic patients, the ones exposed to hepatitis C have a significant higher chance of developing T2D. In the following years, this association was confirmed by other groups and moreover was shown to be a virus-specific observation as hepatitis B virus or other forms of chronic liver disease infection did not associate with T2D [91–93]. Interestingly, this association seems to be bidirectional as T2D patients seem to be more prone to acquire hepatitis C infections [94–96]. Whether or not there is indeed a causative relation between hepatitis C infection and T2D remains to be studied, but deposition of amyloidogenic IAPP is one of the major T2D hallmarks.

Numerous different studies showed correlations between PD symptom development and infection by a certain viral strain, including influenza, West Nile virus, hepatitis C, Japanese encephalitis virus, St. Louis encephalitis virus, coxsackievirus, and Western equine encephalitis virus [88, 97–101]. Most of these did not show direct interactions between viruses and the amyloid form of α-synuclein; however, Bantle et al. [101] recently showed that a neuroinvasive infection of Western equine encephalitis virus (WEEV) induces α-synuclein aggregation in different areas of a mouse brain.

Recently, an analysis of postmortem brain tissue showed a strong correlation between the presence of capsid protein 1 from Ljungan virus (LV), a picornavirus, in neurons and astrocytes, and the development of AD [102]. Moreover, the same authors published a case report in which a small number of patients showed improved or unchanged cognitive function when treated with three different antivirals [102].

## Mechanistic insights into amyloid–virus interactions

Many direct and indirect interactions between human-expressed amyloids and human-infecting viruses have been described today (Table 1). However, relatively little research has been performed to unravel the underlying molecular mechanisms of these interactions. To understand the potential mechanisms of amyloid–virus interactions, it is important to describe how the amyloid aggregation reaction initiates.

**Fig. 1:**
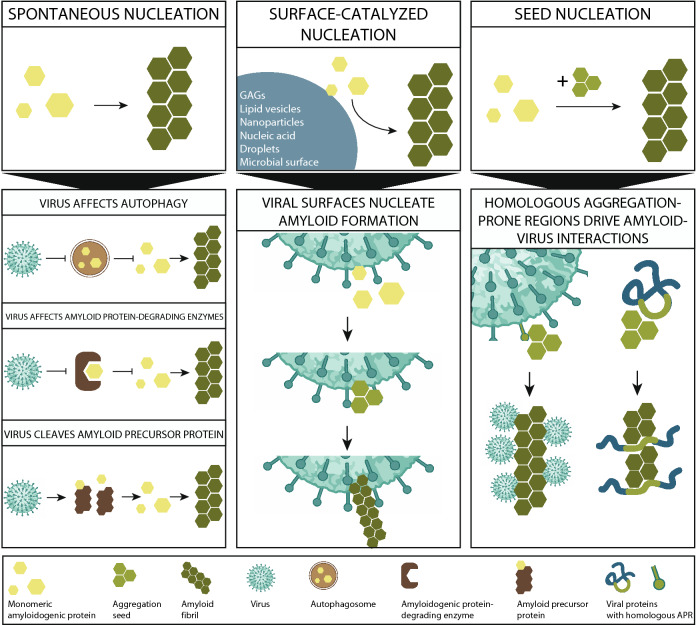
A schematic overview of the mechanisms underlying amyloid–virus interactions. The three upper panels represent three different mechanisms by which amyloid formation can initiate: spontaneous nucleation, surface-catalyzed nucleation (SCN), and seed nucleation (SN). Each of these three mechanisms translates into a different process of amyloid–virus interactions, which are non-mutually exclusive

In its most simple representation, the amyloid reaction can be described as a two-step process [103]. First, an aggregation nucleus or seed is formed, which is the rate-limiting step of the amyloid reaction. Starting from this nucleus, a growth reaction follows in which new monomeric units of a protein are added to the amyloid chain. This two-step nucleation-growth process translates into the typical sigmoidal aggregation reaction including a lag phase (nucleation) and an exponential phase (growth). Hidden in the lag phase, the kinetic description of amyloid aggregation also includes the thermodynamic limitation of nucleus formation: the transition from monomers to an aggregation seed is thermodynamically unfavorable and is therefore a rare event. Providing a preformed aggregate (seed) can promote the aggregation reaction significantly, a mechanism referred to as aggregation seeding. In addition to seeding, other aggregation nucleation events have been described, mainly surface-catalyzed nucleation. To summarize, the amyloid aggregation reaction can be initiated spontaneously or via a catalyzed nucleation event (Fig. 1). These nucleation events can be driven by surfaces in a non-specific manner or by the addition of preformed aggregates (seeding) encoding sequence specificity into the interaction [9, 104].

These three events of amyloid initiation (spontaneous nucleation, surface-catalyzed nucleation (SCN), and sequence-specific seed nucleation (SN)] translate into three non-mutually exclusive hypotheses that have been described to explain amyloid–virus interactions. First, it is possible that viral infections indirectly lead to accumulation and aggregation of amyloidogenic proteins, for example by interfering with degradation pathways of these proteins. Second, some evidence shows that the presence of virion particles might directly spark amyloid formation by surface-catalyzed nucleation. Third, in some cases, sequence-specific amyloid interactions can drive the binding of amyloidogenic proteins or peptides to viral proteins. Here, we summarize the evidence for each of these potential mechanisms underlying amyloid–virus interactions (Fig. 1).

1. Viral infections can indirectly promote spontaneous amyloid nucleation

There are a few events that can lead to spontaneous aggregation, meaning without the addition of a catalyst to initiate the aggregation reaction. First, aggregation-stimulating mutations can include mutations that facilitate spontaneous nucleus formation [105, 106] or complete gene multiplications leading to increased protein levels [107–109]. Second, events leading to an increased local concentration can significantly reduce the nucleation barrier, a prime example being liquid–liquid-phase separation [110]. Third, deterioration of the cellular quality control systems such as chaperones, for example as a result of aging, can lead to increased accumulation of misfolded proteins, also lowering the nucleation barrier for aggregation [111].

In some cases, the latter event has been described to be the driving force of virus-induced amyloid accumulation. For the best-studied case of an amyloid–virus interaction, the association between Aβ and herpesvirus infection, it has been proposed that the dysregulation of autophagy might play an essential role. Herpesviruses interfere with autophagy pathways to optimize their replication and to counteract immune response during primary infection, but also during reactivation from latency [112–117]. For example, gamma herpesviruses block the final steps of autophagy during the lytic cycle for transportation to the membrane [118, 119]. Both HSV-1 and HHV-6B are able to block autophagy in infected neurons and peripheral blood cells, respectively [116, 117, 120]. Remarkably, two other herpesviruses, varicella-zoster virus and HHV-6A, have the opposite effect and promote autophagy to prolong the survival of infected cells [117, 121, 122]. It is well established that cells strongly depend on protein quality control systems to maintain homeostasis. Autophagy is such a mechanism that is used to remove toxic components such as misfolded proteins and protein aggregates, which is especially important for post-mitotic cells like neurons. Shutting down autophagy leads to neurodegeneration and increased neuronal cell death in neurons [123]. One of the characteristics of Alzheimer’s disease is an increased number of autophagosomes in the neurons, suggesting a dysregulation of autophagy [124]. Moreover, aging, one of the most relevant risk factors for amyloid-associated neurodegenerative diseases, is usually accompanied by a progressive reduction of autophagy [125]. More specifically, autophagy is a crucial process in the metabolism of multiple amyloids themselves, including Aβ [126] and tau protein [127]. For example, an autophagy-deficient mouse model showed increased Aβ accumulation and induced neurodegeneration [128]. Altogether, the fact that neurons strongly depend on autophagy and autophagy is involved in the metabolism of the amyloidogenic proteins (Aβ and tau) together with the observation that herpesviruses dysregulate autophagic pathways could indeed suggest a possible indirect link between herpesvirus infection and amyloid-associated pathology in AD.

Additional evidence linking autophagy dysregulation to amyloid–virus interactions resulted from the HIV–Aβ association. It was shown that Tat, the HIV trans-activator of transcription protein, interferes with endolysosome formation [129]. In neurons, Tat induces endolysosome enlargement and disturbs endolysosome function. Additionally, it was shown that intraneuronal Aβ is significantly increased in patients suffering from HIV encephalitis, particularly in autophagosomes [130]. Altogether, these data suggest that the HIV Tat protein might impair endolysosome function and thereby interfere with the proper clearance of Aβ [130].

Also, for the α-synuclein-influenza case, an indirect mechanism affecting autophagy could be the driving force of interaction. Marreiros et al. [50] showed that H1N1 influenza A viral infection and replication leads to a disturbance in protein homeostasis by inhibiting autophagosome–lysosome fusion. The authors show that this leads to impaired α-synuclein degradation and eventually depositions of α-synuclein amyloid.

In addition to the autophagy-mediating effect of viruses, HSV-1 can also influence other upstream events that eventually lead to Aβ and tau amyloid deposition. For example, Civitelli et al. [131] showed that HSV-1 infection in neurons leads to accumulation of APP intracellular domain (AICD), eventually affecting expression levels of NEP and GSK3β. Nep is a major Aβ-degrading enzyme [132, 133], while GSK3β is a serine/threonine kinase that promotes hyperphosphorylation of tau and the increased Aβ production [134]. In this way, HSV-1 could modulate amyloid deposition in an indirect manner.

The HIV Tat protein can also modulate Aβ expression indirectly independent of autophagy [135]. It was shown that HIV infection leads to hypoxia-inducible factor (HIF-1α) up-regulation followed by its binding and inactivation to the long noncoding RNA (lncRNA) BACE1-antisense transcript (BACE1-AS). The latter has been shown to promote the stability of the (sense) BACE1 transcript [136], leading to increased BACE1 levels, the protein responsible for toxic Aβ generation.

Zheng et al. [137] identified an HSV-1-encoded microRNA (miR-H1) that is able to reduce the expression and activity of Ubr1, an RING-type E3 ubiquitin ligase that mediates Aβ degradation. In this way, HSV-1 infection can indirectly lead to the accumulation of Aβ amyloid deposits.

Finally, as mentioned before, Chai et al. showed a direct interaction between the HIV-1 Gag polyprotein and the Aβ precursor protein (APP). Although it is a direct interaction, we prefer to describe it in this section as it was only established as an interaction between one viral protein (not the virion particle) and the precursor protein of Aβ (not the amyloid peptide itself). The authors showed that APP acts as an innate antiviral peptide by sequestering the HIV Gag polyprotein in lipid rafts, in this way blocking further HIV replication [69]. Remarkably, the Gag polyprotein promotes cleavage of APP to escape this antiviral effect of the Aβ precursor protein. This leads to an increased production of the toxic and amyloidogenic Aβ peptide. The authors showed that, as a result of Aβ amyloid formation, primary cortical neurons degenerated, an event that could be prevented by γ-secretase inhibitor treatment.

The evidence described here suggests that upon viral infection, multiple cellular pathways that are associated with amyloid formation or clearance can be disrupted, in this way indirectly affecting amyloid accumulation. However, the opposing effects of some viruses (e.g., HHV-6A promotes, while HHV-6B inhibits autophagy) complicate the understanding of how multiple infections result in similar clinical effects. Thus, although an indirect effect of a viral infection can be the initial spark towards up-regulation of the amyloidogenic protein, most of the evidence described today points towards a direct binding of an amyloid to a viral surface or specific viral protein. As mentioned before, the amyloid aggregation reaction is usually initiated by a catalyzed nucleation mechanism. These can roughly be divided into surface-catalyzed nucleation (SCN) or sequence-specific seed nucleation (SN), and can each explain a direct interaction between amyloids and viruses.

2. Viral surfaces can catalyze amyloid nucleation

It has been known for a while that exogenous surfaces are able to catalyze the amyloid nucleation process, a mechanism referred to as surface-catalyzed nucleation (SCN) [138]. During SCN, a certain surface can increase the local concentration and enables conformational changes necessary to induce the amyloid reaction. Many different surfaces have been shown to lower the energy barrier for amyloid nucleation, including microbial surfaces [10, 101], lipid vesicles [139, 140], nanoparticles [141], glycosaminoglycans (GAGs) [142, 143], nucleic acids [144] and even the surface of a liquid–liquid-phase separation droplet [145]. A special case of SCN is secondary nucleation, in which the surface of a preformed aggregate, instead of the reactive ends, serves as a catalytic spot for amyloid initiation [146].

Recently, a few studies point towards SCN as a crucial factor in virus-induced amyloid depositions. Starting with the best-studied case of the Aβ-herpesvirus interaction, co-localization of HSV-1 DNA in amyloid plaques was a first indication of a direct interaction between the two. In 2015, Bourgade et al. [60] showed that Aβ inhibits HSV-1 replication by a direct binding event to its surface glycoproteins. Moreover, they showed that Aβ only exhibits this antiviral effect when the amyloid is added before the virus, indicating a direct extracellular event on the virion particle. Indeed, Aβ did not affect the replication of a non-enveloped human adenovirus and the antiviral effect was removed by simply washing away the extracellular Aβ.

Building on these findings, Eimer et al. [6] showed that this binding event of Aβ oligomers onto the surface of HSV-1 particles induces Aβ fibril formation. This event accelerated Aβ plaque formation in 5XFAD transgenic mice and at the same time protected those mice from a harmful HSV-1 infection. Of note, the authors showed the same effect for two other herpesviruses (HHV-6A and HHV-6B) in a 3D human neural cell culture infection model. The authors attributed this effect to SCN on microbial surfaces. They show that the seeding propensity of virion particles is 1–2 orders of magnitude more rapid than reported for Aβ fibrilization mediated by host glycosaminoglycans. They hypothesize that microbial sugars, rather than host glycosaminoglycans, are the actual target of Aβ oligomers.

Ezzat et al. [10] performed a more unbiased analysis of the proteins binding to the surfaces of RSV and HSV-1, termed the viral protein corona. Interestingly, they show that each virus composes a unique protein corona and that the specific protein layer determines the infectivity of the virus. More specifically, they showed that amyloidogenic peptides including IAPP and Aβ were able to bind the surface of RSV and HSV-1, respectively. Even more, the virion particles were able to nucleate the amyloid formation of both peptides in vitro and in animal models, confirming the observations made by Eimer et al. [6].

In addition to the HSV-1 case, it was also shown that Aβ can induce the aggregation of influenza A virion particles by physical interactions [70], indicating that a similar mechanism is at play in this case.

Another direct interaction between virion particles and amyloids was observed between HIV and SEVI amyloids [8]. Amyloids formed by a short peptide derived from PAP are present in the semen of healthy individuals [74] and upon HIV infection bind to the virion surface. This binding event, driven by the cationic properties of the fibrils, drastically enhances virion attachment to and fusion with target cells [147]. Removing the positive charges, but maintaining the amyloid propensity, did abolish the promoting effect on viral infectivity. The latter suggests that the positively charged amyloid fibrils allow the viruses to come into close proximity to the negatively charged host membrane and in this way facilitate infection. This was confirmed by the fact that the infection enhancing effect was not observed for Aβ fibrils. However, Aβ fibrils have been shown to bind negatively charged phospholipid membranes [148]. Additional amyloids, for example those formed by the bacterial curli proteins (Csg A and CgsB), also do not increase HIV infectivity [89]. Interestingly, an HIV gp120-derived peptide produced from the natural degradation in gp120-loaded rat hepatocytes induced the aggregation of SEVI [149]. This finding suggests that although SEVI amyloid is present in healthy individuals, HIV virion particles might boost the stimulation of increased SEVI amyloid levels to increase its own infectivity. Later, Chen et al. [150] showed that the gp120-derived peptide can also self-assemble into amyloid fibrils and enhance HIV infectivity without SEVI.

For the association between HIV and Aβ, a very specific interaction effect has been described [151]. Using in vitro studies, the authors showed that when the HIV Tat protein was added to preformed Aβ amyloid fibrils, these fibrils matured into thicker, unstructured fibrils. This lateral reorganization resulted in fibrils with increased rigidity and mechanical resistance. Moreover, Tat protein and Aβ exerted a synergistic neurotoxic effect both in vitro and in vivo. The authors hypothesized that the reorganized fibrils may account for increased neuronal damage due to increased potential to form transmembrane pores.

3. Sequence-specific nucleation can drive amyloid–virus interactions

In contrast to SCN, the best-studied case of aggregation seeding is homologous seeding: a preformed aggregate (seed) of the same protein can act as a template to initiate the aggregation of more monomeric protein. Almost two decades ago, it was shown that these aggregation seeding reactions strongly depend on similarity of their sequences: preformed aggregates most efficiently induce the aggregation of proteins encoding high sequence similarity, a process referred to as homologous seeding [23–26]. This sequence dependence originates from the underlying molecular structure of amyloid aggregates: the tight packing does not easily allow sequence mismatches in the core. However, on multiple occasions, it has been shown that incorporation of sequences that are only slightly different can still fit into the cross-β steric zipper [25] and this is referred to as heterotypic interactions (or cross-seeding).

**Fig. 2:**
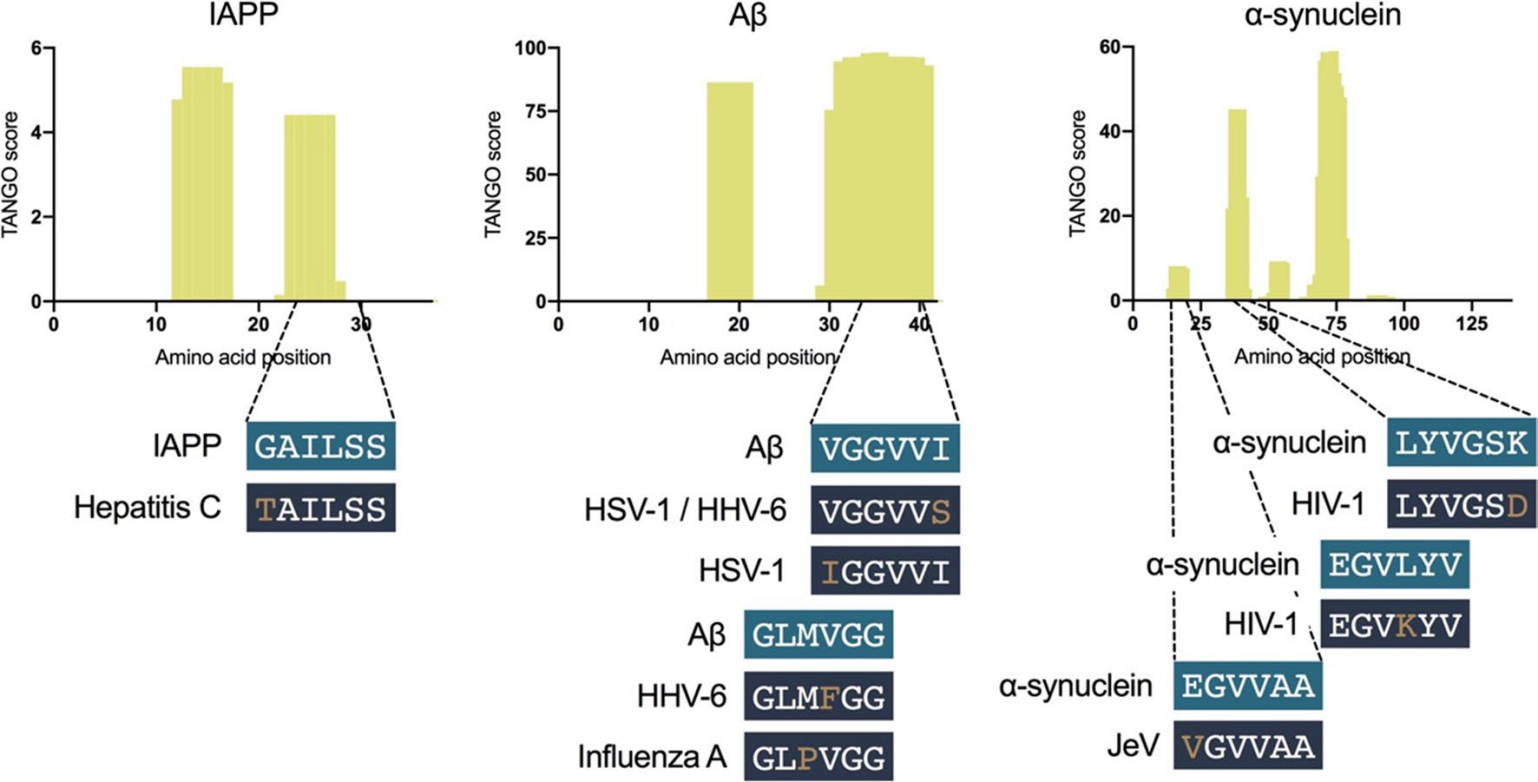
Human-expressed amyloids share homologous APRs with viral proteins. The graphs represent TANGO analyses to identify APRs in the amyloids [17]. The TANGO score is a value between 0 and 100 and higher scores represent higher aggregation potential of the amino acid sequence. As such, the yellow bars represent the APRs of the amyloids. Below each graph, amyloid hexamer APR sequences are visualized that share a homologous fragment within a viral protein. Only homologous sequences with one amino acid mismatch are shown

Some of the amyloid–virus interactions that have been described so far point to a sequence-specific amyloid interaction. If a certain amyloidogenic peptide shares a homologous amino acid stretch with a viral protein, this allows the initial interaction between the two that sparks the amyloid fibrillation reaction. More specifically, if viral surface proteins encode such homologous stretches to amyloid-prone peptides, some of the interactions that are now thought of as surface-induced could potentially have a sequence-specific driving force. Already 2 decades ago, it was pointed out that the glycoprotein B (gB) of HSV-1 shares a homologous region with Aβ, with the highest degree of similarity located at the N-terminal amyloidogenic region of Aβ (Fig. 2) [152]. Even earlier, it was suggested that the same region of gB is responsible for neurotoxicity [153] and fibril formation [154]. In the study published by Eimer et al. [6], the importance of this viral glycoprotein was highlighted by the fact that the interaction between HSV-1 and Aβ could be inhibited by blocking these viral surface proteins. Moreover, a scrambled version of Aβ did not interact with virion particles, highlighting the potential sequence specificity of this interaction [6, 60].

Glycoprotein B is not the only surface protein that shares a homologous sequence to Aβ: a 28-residue-long peptide derived from HSV-1 glycoprotein K (gK) organizes into amyloid fibrils and shares a very similar region with a six-amino acid stretch in the N-terminal amyloidogenic region of Aβ (gK, VGLIVG; Aβ, IGLMVG). Interestingly, this peptide is expected to result from cleavage by the human 20S proteasome [155].

Looking back at the study performed by Ezzat et al. [10], both RSV and HSV-1 compiled a unique protein corona on their surface and remarkably, both viruses induced the aggregation of unique amyloidogenic fragments. For example, RSV induced the amyloid fibrillation of an IAPP peptide. This seeding event was abolished by pre-incubation of the virion particles in a 5% serum solution, indicating that a physical interaction with the virion surface or specific viral surface proteins is crucial to induce amyloid fibrillation. Of pivotal importance, however, is the fact that another amyloidogenic peptide (GNNQQNY), derived from the yeast Sup35 prion protein, was not seeded by the same virion particles in identical conditions. The latter indicates that sequence-specific interactions might underlie the initial binding between amyloidogenic peptides and viral surface proteins. This study, however, also indicates that the amyloid-interacting protein on the viral surface is not necessarily a viral protein, as viruses sequester a unique and diverse host protein corona. Even though RSV primarily infects the respiratory system, RSV virion particles have been detected in human myocardial tissue, liver, and cerebrospinal fluid [156], where it could physically interact with IAPP, a peptide hormone. Of note, Ezzat et al. also confirmed previous findings that HSV-1 virion particles seed the amyloid aggregation of Aβ.

In the case of the IAPP–VZV interaction, it was shown that VZV-infected cells show an increased intracellular IAPP amyloid load. Moreover, the supernatant from VZV-infected cells induced IAPP aggregation. It was shown that preformed seeds formed by peptides derived from VZV glycoprotein B could induce IAPP aggregation [72]. The latter is an example of a typical amyloid seeding event and could explain the induced IAPP aggregation upon VZV infection.

Also for the HIV case, homologous sequence segments with Aβ have been described. It was shown that a region from the HIV gp120 protein (residues 24–28 in a typical V3 loop, GAIIG) self‐assembles into amyloid fibrils [157] and shares an identical stretch with Aβ. Importantly, no interaction studies between the two fragments were performed in this study.

Therefore, it is possible that at least for some cases, the interaction between amyloids and viruses is driven by homologous sequences that induce an amyloid-like interaction. Moreover, such interactions are not limited to the viral surface, as is the case for viral surface-catalyzed nucleation, but can also include interactions between amyloids and viral non-surface proteins. For example, regarding the known interaction between influenza A virion particles and Aβ [70], it was shown that the C-terminal fragment of Aβ is the driving force [71]. This amyloidogenic C-terminal fragment of Aβ shares a homologous region with a viral non-surface protein, the RNA-directed RNA polymerase (Fig. 2). Physical interactions between the amyloidogenic peptide and the viral protein would then be possible inside the virion particle or inside the infected host cell.

Figure 2 visualizes homologous APRs sequences between human amyloids and viral proteins. Whether or not such homologous sequences drive their interaction and a subsequent amyloid seeding event remains to be studied, but the presence of this sequence similarity at least shows the possibility of a co-evolution event. Amyloids could have evolved as a natural defense mechanism to protect against the acute threat of a viral infection. Their subsequent accumulation in human tissue could then be an unwanted side-effect of their antimicrobial activity and usually only causes problems years after aggregation initiation.

## Therapeutic potential

**Fig. 3:**
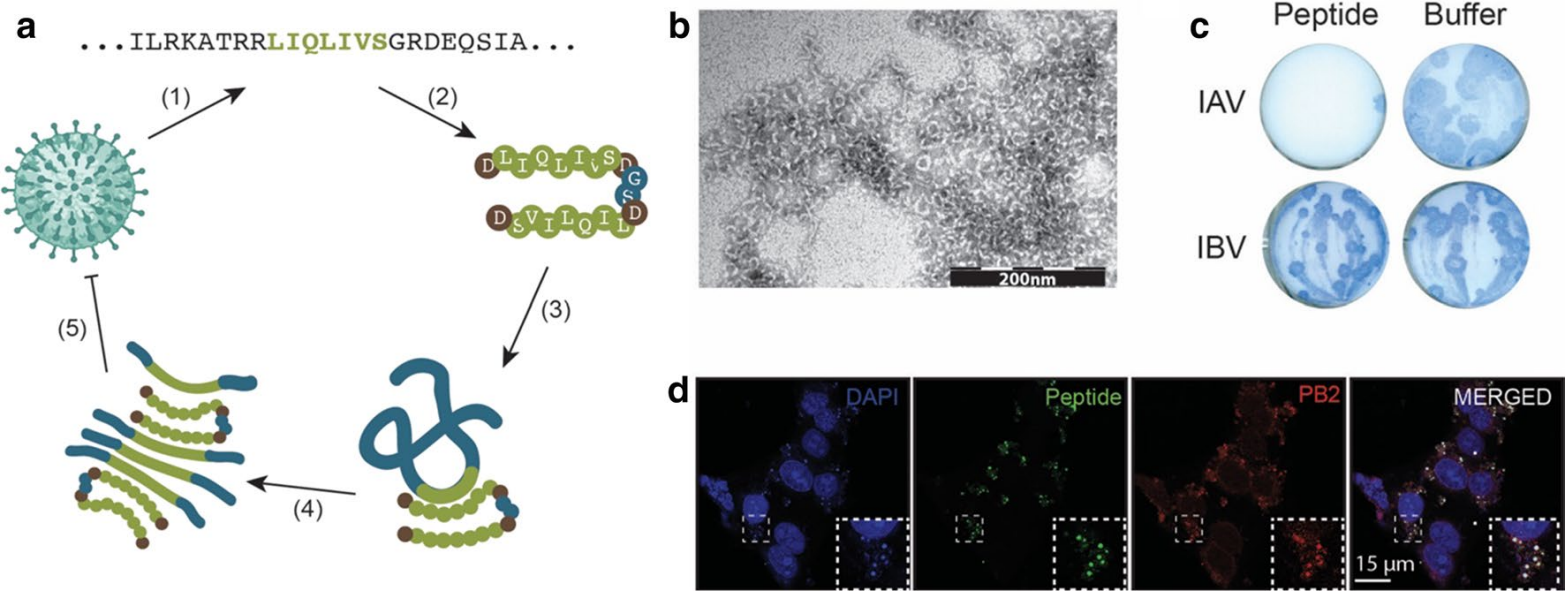
Design of synthetic antiviral amyloids solely based on homologous APRs. (a) A schematic overview of the design principle of synthetic antiviral amyloids: (1) APRs are identified in the proteome of a specific virus (here: LIQLIVS from the influenza A/PR8 PB2 protein), (2) peptides are designed based on this APR sequence in a tandem design format to stimulate amyloid formation, (3) this synthetic amyloidogenic peptide can interact with the viral target protein via the homologous APR sequence, (4) which leads to the aggregation and inactivation of that protein, and (5) finally reducing viral replication. **b** Incubating the peptide visualized in **a** for 1 h at 100 µM results in amyloid-like structures. **c** A plaque-size reduction assays showing that the amyloidogenic peptide (10 µM) inhibits influenza A (IAV) replication (blue area), while influenza B (IBV) is not affected. **d** Colocalisation of a fluorescent variant of the amyloidogenic peptide (10 µM) and PB2 in influenza A-infected MDCK cells. All data were taken and adjusted with permission from [158]

These data indicate that homologous aggregation-prone regions between an amyloidogenic peptide and a viral protein can drive their interaction. We recently developed a fully synthetic amyloid that allows a specific interaction with an influenza polymerase protein: polymerase basic protein 2 (PB2) [158]. The synthetic peptide is not related to Aβ or any other human-expressed amyloid, but shares a homologous fragment with PB2. We have shown that this synthetic peptide organizes into amyloid-like structures, enters infected host cells, specifically binds to the influenza A PB2 protein, and induces its aggregation (Fig. 3). The interaction between the synthetic amyloid and PB2 is driven by their homologous fragment, as no interaction is observed between the influenza B PB2 protein, which lacks that fragment, or between a mutated synthetic amyloid and the influenza A PB2 protein (Fig. 3c). The latter is a crucial observation, since the synthetic amyloids should also not induce the aggregation of any host proteins. The sequence specificity of aggregation seeding reactions as described earlier allows to specifically target viral proteins if a similar amino acid stretch is not present in the host proteome. The induced aggregation of the influenza A PB2 proteins leads to a reduction of viral replication and as such the amyloid exerts an antiviral effect. As a similar antiviral effect was observed when the amyloid was added before or after viral infection, and pre-incubation of the virion particles with the amyloid did not increase the efficacy, this suggests that the amyloid–viral protein interaction occurs inside the infected host cell. This finding was confirmed by the colocalisation of the synthetic amyloid and the PB2 target protein in the cytoplasm of infected cells (Fig. 3d). Moreover, the synthetic amyloid accumulated only in influenza A-infected tissue in vivo, while no amyloid deposition was observed in non-infected tissue.

The same approach was used to design a second, unrelated amyloid that specifically binds to a Zika virus surface protein: membrane glycoprotein M. This synthetic amyloid showed specific antiviral activity on Zika virus particles. and proved that this approach can be used to target both surface and non-surface viral proteins [158].

Zhang et al. recently showed that such approach can also be used as a detection tool for viral infections [159]. The authors designed a short amyloid-forming peptide, whose sequence is based on an aggregation-prone region identified in an SARS-CoV protein, S-protein. This amyloidogenic peptide is able to bind to the S-protein via the homologous region and, in this way, can be used as a highly specific detection tool for SARS-CoV infection [159].

The data described here show that the self-assembling properties of amyloidogenic peptides can be used as biochemical or therapeutic tool. Moreover, as in some cases of naturally occurring amyloid–virus interactions, such similar amino acid stretches are indeed present, it is possible that they are as well regulated by the same underlying mechanism that drives amyloid reactions. Such interactions are not limited to viral surface proteins as amyloid interactions can occur inside the infected host cell.

## Conclusions

About 35 peptides and proteins are known to organize into amyloid that are associated with human diseases and it is estimated that protein aggregation affects some 500 million people worldwide [9, 160]. Intensive research has shed some light on the molecular mechanisms underlying amyloid formation; however, a comprehensive understanding of their initiation and toxicity in disease remains incomplete. It has been shown that multiple (disease-associated) amyloids encode important cellular functions [4, 5], including as part of the host’s innate immunity against microbial pathogens. Indeed, multiple amyloids have been shown to reduce viral replication in vitro and in vivo [6, 69, 70], both indirectly, via autophagy or interference with specific host protein expression levels, and directly, via binding to viral (surface) proteins. It has been proposed that cells evolved to express amyloidogenic peptides or proteins as a first line of defense mechanism to reduce the acute treatment of a viral infection. A viral infection could then be the initial trigger for amyloid accumulation, which leads to the pathogenic side-effects, usually, decades after amyloid accumulation initiated. However, it must be mentioned that although viral infections are common in many amyloid-associated diseases, amyloid formation in these diseases can also be sparked by other phenomena, including mutations or deterioration of the PQC system as a result of aging.

Crucially, this antiviral effect of amyloids is not universal as semen-derived amyloids seem to exert the exact opposite effect: they stimulate HIV and Ebola infections. It is important to note that, in this case, the amyloids are also present in healthy, non-infected people, so they are not expressed as a reaction to viral infection. This confirms findings that amyloids are not intrinsically toxic [3] but also raises the question of whether those amyloids encode different functions in addition to assisting infections of pathogenic microbes. Viruses very often depend on multiple host factors for infectivity, the replication of their genetic material or virion assembly, so it is not unlikely that HIV and Ebola viruses have evolved to employ host amyloids to promote their infectivity. Either way, it is currently not known why some amyloids stimulate viral infections and others inhibit their replication. A systematic study of the effect of different amyloids, both disease-associated and not, on viral infectivity could help resolve this question.

Here, we summarized the known interactions between human amyloids and human-infecting viruses and focused on the potential underlying mechanism driving these interactions. We conclude that so far, three different hypotheses have been suggested and we aim to connect these hypotheses to the well-studied mechanisms of amyloid initiation: spontaneous nucleation, SCN, and sequence-specific SN. The three mechanisms of amyloid–virus interactions described here are non-mutually exclusive: viral infection can induce the up-regulation of amyloidogenic proteins or interfere with their degradation, followed by a seeding event on virion surface proteins. Once the aggregation has initiated, soluble oligomeric seeds can specifically bind to viral proteins that express a homologous APR, and in this way, amyloids can interact with viruses in a sequence-specific manner. Whether or not such mechanisms of interaction are indeed broadly used for amyloid virus interactions remains to be determined, but homologous APRs between amyloids and interacting viral proteins have been described. Moreover, we have shown that such interactions can be used in a synthetic biology approach to design antiviral amyloids [158]. By reverse-engineering synthetic amyloids that encode a specific viral APR, these amyloids are able to bind to the corresponding viral protein and induce its aggregation. Interestingly, such observations have been made in a physiological context, as well: the herpesvirus protein M45 induces aggregation of two host proteins, NEMO and RIPK1, to block innate antiviral responses [161]. A specific 5-amino acid motif in the C-terminal part of M45 protein recruits the two host proteins and induces their aggregation. The M45-induced sequestration of NEMO and RIPK1 subsequently facilitates their degradation by autophagy. Whether or not this motif engages in an amyloid-like interaction with the host proteins remains to be determined, but in this case, virus-induced host protein aggregation promotes viral infectivity. Of note, this motif acts as a traditional APR as fusion the motif to mCherry is sufficient to induce mCherry aggregation.

Interestingly, multiple viruses have evolved to express amyloid-forming proteins to increase virulence. Rift Valley fever virus (RVFV) expresses a protein called NSs that rapidly organizes into large filamentous structures in the nuclei and to a lesser extent in the cytosol of infected cells. These filaments show the characteristic features of amyloids [162] and have been linked to RVFV virulence. Mice infected with RVFV lacking NSs survive, while those infected with wt RVFV die within a few days as the result of severe encephalitis. Léger et al. [162] showed that the virus employs NSs amyloid formation to suppress IFN response to counteract host cell defenses. Moreover, NSs amyloid fibrils were identified in the neurons of intraperitoneally infected mice and it was shown that the NSs are the causative agent for neuropathy.

Unraveling the potential role of disease-associated amyloids in innate immunity against invading pathogens could shed more light on the pathogenic effect of these amyloids and provide new therapeutic opportunities. General antiviral treatments in AD patients already showed some promising effects, but a more specific approach will be needed to obtain conclusive results. Moreover, if amyloids, indeed, encode specific antiviral properties, together with the fact that most amyloids are not intrinsically toxic [3], synthetic or natural amyloids could be used as a new class of antimicrobial peptides (AMPs) [158]. Indeed, the molecular architecture of amyloid structures entails the ability to engage in very specific interactions with other proteins, affecting their biological function.
